# Supplementary material for: Transition from hospital to home care for preterm babies: A qualitative study of the experiences of caregivers in Uganda
Source: PLOS Glob Public Health. 2023 May 8;3(5):e0000528. doi: 10.1371/journal.pgph.0000528 (PMC10166523; doi:10.1371/journal.pgph.0000528)
Supplement: Attachment — Submitted filename: Response to reviewers.docx [file pgph.0000528.s002.docx]

**Response to reviewer’s comments**

**Addressing Journal requirements**

1. Please review your reference list to ensure that it is complete and correct.
   **We have reviewed the reference list and can confirm that we do not have any retracted papers in the reference list. The list is complete and correct to the best of our knowledge.**
2. Please include a complete copy of PLOS’ questionnaire on inclusivity in global research in your revised manuscript.
   **The filled questionnaire is included as supplementary material, as advised.**

**Comments to the Author**

1. Does this manuscript meet PLOS Global Public Health’s [publication criteria](https://journals.plos.org/globalpublichealth/s/criteria-for-publication)? Is the manuscript technically sound, and do the data support the conclusions? The manuscript must describe methodologically and ethically rigorous research with conclusions that are appropriately drawn based on the data presented.

Reviewer #1: Yes

2. Has the statistical analysis been performed appropriately and rigorously?

Reviewer #1: N/A

3. Have the authors made all data underlying the findings in their manuscript fully available (please refer to the Data Availability Statement at the start of the manuscript PDF file)?

The [PLOS Data policy](https://journals.plos.org/globalpublichealth/s/data-availability) requires authors to make all data underlying the findings described in their manuscript fully available without restriction, with rare exception. The data should be provided as part of the manuscript or its supporting information, or deposited to a public repository. For example, in addition to summary statistics, the data points behind means, medians and variance measures should be available. If there are restrictions on publicly sharing data—e.g. participant privacy or use of data from a third party—those must be specified.

Reviewer #1: Yes

4. Is the manuscript presented in an intelligible fashion and written in standard English?

PLOS Global Public Health does not copyedit accepted manuscripts, so the language in submitted articles must be clear, correct, and unambiguous. Any typographical or grammatical errors should be corrected at revision, so please note any specific errors here.

Reviewer #1: Yes

5. Review Comments to the Author

Reviewer #1: Congratulations to the authors for a beautiful manuscript! This manuscript describes the results of a qualitative study of 56 caregivers of preterm infants in Uganda through 7 FDDs and 5 IDIs. This manuscript is very well-written and provides excellent information about the the challenges experienced by caregivers of preterm infants in Uganda while transitioning from hospital to home care in order to identify the needs, opportunities and potential interventions for improving support systems. The introduction is well organized; the methods and analysis section is very clear and complete.

**Response:** We thank the reviewer for the positive and encouraging comments.

I have only a few comments that could strengthen the results and discussion sections:
Results:
Page 9, lines 186-191
I am not sure the quotes fully support and/or link the statement "their newly born preterm baby because they looked “small” and fragile." to lack of acceptance or denial among parents. The quotes don't really convey the lack of acceptance or denial among parents. Just because the parents expressed fear, it doesn't mean that they are denying their preterm infant. Perhaps the challenge here is to distinguish the pregnancy/birthing process from the actual infant?

**Response:** We agree with the reviewer and have revised the manuscript accordingly. Please see page 9, lines 186-191 in the revised manuscript with track changes.

Discussion
Page 20, line 446-448: Either here or in the relevant place in the results, it would be interesting to include specific drugs or medicines that were identified by parents as being costly to procure and that weren't provided free of charge for the health system.

**Response:** We thank the reviewer for the comment. By essential medicines, the parents/caregivers meant essential micronutrient supplements and prophylaxis for anaemia in preterm infants such as multivitamin syrup and haematinics. We have now clarified this in the results section, page 11, lines 230-232, and in the discussion section on page 20, lines 456-457 in the revised manuscript with track changes.

Rudeness of the providers is documented in the literature although not specifically when related to caring for preterm infants. It could strengthen the discussion section to include one or two of such articles and related points.

**Response:** As advised by the reviewer, we have added this point with references in the discussion section on page 19, lines 423-427 in the revised manuscript with track changes.

Link the comments about "counsellors" on page 11, line 223 to the discussion about peer support more directly.

**Response:** As advised by the reviewer, we have linked the comment about "counsellors" in the results section more explicitly and directly with the discussion about peer support on page 20, lines 446-447 in the revised manuscript with track changes.

Page 21, line 480-482: I don't agree with the claim made by the authors that they included perspectives of caregivers whose infants had died by interviewing a parent of twins where one of the twins had died. Because the experience of having a preterm infant who died is significantly different, I suggest revising this statement to say that a limitation of the study is that the lived experience of parents whose infants died were not included.

**Response:** We agree with the reviewer and have deleted the sentence “However, we interviewed a parent of twins where one of the twins had died, and in this way, offer some insights into such experiences.” Please see page 22, lines 491-493 in the revised manuscript with track changes.

As advised, we have added the following sentence on page 22, lines 487-488 in the revised manuscript with track changes.

“However, a limitation of the study is that lived experiences of parents whose infants died were not included.”
