# Supplementary material for: Transition from hospital to home care for preterm babies: A qualitative study of the experiences of caregivers in Uganda
Source: PLOS Glob Public Health. 2023 May 8;3(5):e0000528. doi: 10.1371/journal.pgph.0000528 (PMC10166523; doi:10.1371/journal.pgph.0000528)
Supplement: Attachment — Submitted filename: Response to reviewers comments_R2.docx [file pgph.0000528.s003.docx]

**Response to reviewer’s comments**

Review Comments to the Author

Reviewer #1: (No Response)

Reviewer #2: Dear Editor,
This submission presents careful qualitative research on the experience of people with the subject of preterm babies. I believe it makes a useful contribution. The English is good overall; however, the manuscript still requires some improvements in places. I have made some suggestions in the following comments.

Response: We thank the reviewer for the positive comments.

Method section:
Page4 lines 95-96
o The authors mentioned to “comprehensive preterm care system” in two cities in different hospital settings”. I recommend providing more details on this system and why they call it comprehensive. Also, what is the difference between a general hospital and a regional referral hospital?

Response: As advised by the reviewer, we have explained what we meant by comprehensive preterm care provided in Iganga hospital. We have also explained what a regional referral hospital is in the revised manuscript, (please see page 5, lines 103-106 in the manuscript with track changes).
To answer the reviewer’s question, a regional referral hospital has a wider catchment area than a general hospital as patients from a wider geographical region are referred by smaller local hospitals for treatment.

Page5 line 100
o Please define “critically ill” preterm infants that you were excluded from the study.

Response: As advised, we have described what we meant by “critically ill” infants in the revised manuscript (page 5, line 111 in the manuscript with track changes).

Page5 line3 97-110
o The authors mentioned “Our study population included caregivers of preterm infants defined as parents (mothers and fathers), or other relatives or carers.”. however they did not mention how they balance these participants in the study. Did they determine the number of participants in each group of caregivers? If carers were from hospital personnel then it could make bias during the focus group discussion.

Response: We thank the reviewer for raising two valid points. Although our sampling strategy was informed by maximum variation, we did not aim to balance the number of fathers, mothers and other caregivers as this would make the study extensive and not practically feasible. We have clarified this in the revised draft (page 5, lines 119-122 in the manuscript with track changes).

We agree with the reviewer that if the carers were selected by the hospital staff, they might be influenced during focus group discussions leading to bias. We would like to clarify that hospital staff did not directly select the carers, instead provided a list of eligible participants and their contact address. The study team selected and approached the participants from the list using purposive sampling to attain maximum variation (page 6, lines 124-127 in the manuscript with track changes).

Result section:
o I recommend making a table from the main themes/codes in the result section and mentioning the important points of each theme/code in the table.

Response: As advised by the reviewer, we have added a new Table-2 with a summary of the themes and sub-themes with key points. Please see pages 19-21, lines 421-422 in the manuscript with track changes.

Conclusion section:
o This part does not need to use references. Comparing the results with other studies should mention in the discussion section.

Response: As advised by the reviewer, we have moved the following sentence from the conclusion to the discussion section – “However, while there are several studies evaluating the benefits of peer-to-peer support for parents in the NICU [32-37], evidence about the effectiveness of such support at home is limited [6].” Please see page 23, lines 486-488 in the revised manuscript in track changes.

Reviewer #3: I would like to congratulate the authors for the beautiful work! This manuscript describes the experiences of 56 caregivers of pre-term babies while transition from hospital to home-care in Uganda and aims to identify the needs, opportunities and potential interventions for improving support systems. The introduction is well written; the methods, especially of conducting thematic analysis are appreciable and the results are systematic. The hard-work put by all the authors in the manuscript is commendable.
I have few comments and suggestions, which might be helpful to strengthen the manuscript:

Response: We thank the reviewer for the positive comments.

1. Can you please explain in the methods section that how many participants were selected from each hospital? Was the sample drawn proportionate to the number of pre-term births happened at both the hospitals or just convenience sampling was done? As in the inclusion criteria, parents of 1-12 months aged infants are taken and in the SDCs table 1, there are two age- groups of infants, but caregivers of 1-2 months babies are not there, did that happen by chance?

Response: We have explained in the ‘sample size and sampling method section, how the participants were selected, “We conducted seven FGDs (each with 6-8 participants) and five IDIs among a purposively selected sample of caregivers of preterm infants.”. Please see page 5, lines 114-115 in the revised manuscript in track changes. Based on comments from Reviewer-2 & 3, we have further clarified that the selection process was purposive sampling to attain maximum variation (page 5 lines 119-122, revised manuscript with track changes). We did not use convenience sampling.

The reviewer has raised a good point about our inability to include caregivers of preterm infants aged 1-2 months. This was not by chance, but we had difficulty in recruiting caregivers of very young infants who were unable to give time. We have now acknowledged this in the limitations section on page 25, lines 520-522 in the revised manuscript with track changes.

2. For pre-testing FGD guide, how did you select the participants? It is mentioned that caregivers from different localities were selected. Was the hospital data used to identify them or some other method used?

Response: As advised by the Reviewer, we have explained that the participants for pre-testing the guide were selected from the hospital discharge records (page 6 lines 130-131, revised manuscript with track changes)

3. Infants may have different requirements or needs at different age; for example, KMC can be crucial for younger babies than their older counterparts. So, I am curious to know that were there any different criteria/questions to interview the caregivers of different aged infants, or have you recorded any typical responses from them?

Response: We thank the review for the question. We did not use different criteria/questions for interviewing caregivers of younger and older babies. We explored caregivers’ experiences of caring for the baby since birth. We did not undertake separate analysis based on the age group of the infants as this was not within the scope of our research and we did not specifically collect information to explore this. Our aim, as mentioned in the introduction section, was to understand the challenges experienced by caregivers of preterm infants in Uganda while transitioning from hospital to home care.

4. Initially it has been mentioned that healthcare providers were very supportive (line no. 177), but later it is stated that they were not empathetic (line no.238) and were rude. So these are contradicting statements, please explain or resolve it.

Response: We understand that the two points might appear conflicting. However, as explained on page 10, lines 194-195, “Although the caregivers largely felt supported by healthcare workers while in the hospital, there were questions about appropriate communication”. We have further edited the results section to make this clearer. Please see page 12 lines 255-259 of the revised manuscript in track changes.

5. With due respect, if possible, please give some explanation in the introduction section about stigma of having or experiencing pre-term babies in the study population. As a reader it feels little awkward to read so many times that people felt disgusted about the pre-term baby or hated them. If some background is set explaining such behavior, it will be easier for readers from other cultures to understand.

Response: We thank the Reviewer for raising a valid point. We have added information in the introduction section from other published literature from sub-Saharan Africa about stigma related to preterm infants and small babies. Please see page 3, lines 64-70 in the revised manuscript in track changes.

6. The title of the study though only depicts experiences of the caregivers during the transition, but aim of the paper is broad and intends to identify potential interventions to improve support system. I have felt that the authors should elaborate the proposed interventions which are also practical in that region, some more explanation should be given towards the last part of discussion section which justifies the aim.

Response: We would like to clarify that while peer-support was mentioned by the participants and explored in the study, our aim was not to identify potential interventions to improve support system, which would be a separate study. However, as advised by the reviewer, we have added some more explanation in the last part of the discussion section, specifically mentioning that peer-support could be a potential intervention. Please see page 24, lines 492-498 in the revised manuscript with track changes.

7. Can you give a reference to the lines 353-355 (peer-support).

Response: As these lines are in the results section, we did not add a reference here, but we agree with the reviewer that it is important to describe the concept of peer-support. Therefore, we have added a description in the introduction section itself. Please see page 4, lines 79-81 in the revised manuscript with track changes.

Reviewer #4: (No Response)
